# Two Patterns of White Matter Connection in Multiple Gliomas: Evidence from Probabilistic Fiber Tracking

**DOI:** 10.3390/jcm11133693

**Published:** 2022-06-27

**Authors:** Simin Zhang, Xiaorui Su, Graham J. Kemp, Xibiao Yang, Xinyue Wan, Qiaoyue Tan, Qiang Yue, Qiyong Gong

**Affiliations:** 1 Huaxi MR Research Center (HMRRC), Department of Radiology, West China Hospital of Sichuan University, Chengdu 610041, China; 18708139153@163.com (S.Z.); xiaorui_su531@163.com (X.S.); awan1204@163.com (X.W.); 2 Huaxi Glioma Center, West China Hospital of Sichuan University, Chengdu 610041, China; yang061528@163.com; 3 Liverpool Magnetic Resonance Imaging Centre (LiMRIC), Institute of Life Course and Medical Sciences, University of Liverpool, Liverpool L69 3GL, UK; g.j.kemp@liverpool.ac.uk; 4 Department of Radiology, West China Hospital of Sichuan University, Chengdu 610041, China; 5 Department of Radiotherapy, West China Hospital of Sichuan University, Chengdu 610041, China; 15513037138@163.com; 6 Department of Radiology, West China Xiamen Hospital of Sichuan University, Xiamen 361021, China

**Keywords:** multiple gliomas, diffusion tensor imaging, probabilistic fiber tracking, white matter connection, cluster analysis

## Abstract

Background: Multiple lesions are uncommon in brain gliomas, and their pathophysiology is poorly understood. Invasive growth along white matter tracts is an important clinicopathological characteristic of gliomas, and a major factor in a poor therapeutic outcome. Here, we used probabilistic fiber tracking and cluster analysis to investigate the inter-focal connectivity relationships of multiple gliomas, in order to seek inferential evidence of common origin. Methods: MRI scans of 46 patients with multiple gliomas were retrospectively analyzed. Before surgery, all patients underwent multimodal functional MR imaging, including diffusion tensor imaging, enhanced 3D T1-weighted imaging, diffusion-weighted imaging, 1H MR spectroscopy, and dynamic susceptibility contrast perfusion-weighted imaging. Probabilistic fiber tracking was used to quantify white matter connectivity between neoplastic foci. Hierarchical cluster analysis was performed to identify patterns of white matter connection. Results: Cluster analysis reveals two patterns of connectivity, one with smaller, and one with greater, connectivity (2675 ± 1098 versus 30432 ± 22707, *p* < 0.0001). The two subgroups show significant differences in relative cerebral blood volume (2.31 ± 0.95 versus 1.73 ± 0.48, *p* = 0.002) and lipid/creatine ratio (0.32 ± 0.22 versus 0.060 ± 0.051, *p* = 0.006). Conclusion: Two distinct patterns of white matter connection exist in multiple gliomas. Those with lower connectivity tend to have independent origins, and can be termed true multicentric glioma, whereas those with greater connectivity tend to share common origin, and spread along white matter tracts. True multicentric gliomas have higher vascularity and more intratumoral necrosis. These findings may help to develop personalized therapeutic strategies for multiple gliomas.

## 1. Introduction

Multiple gliomas, characterized by the presence of two or more neoplastic foci, are rare (2–9% of all gliomas [1,2,3]), but because of the higher disease burden [4], genetically more aggressive phenotype [5], and unsuitability for gross tumor resection, they are associated with worse clinical outcomes and poorer survival rates than solitary gliomas [6,7].

The pathogenesis of multiple gliomas is not fully understood. Three genomic analyses strikingly reveal that foci from the same patient were of monoclonal origin [8,9,10]. By analogy with glioblastoma recurrence following treatment [11], this seems to imply spatial continuity between the different foci, due to invasion of the whole brain by lower grade cells, which undergo malignant transformation at separate points. However, other studies suggest that different neoplastic foci develop simultaneously and independently [12,13]. Thus, the results of genetic analysis are still controversial.

Taking a different approach, some argue that if there is a microscopic connection between the foci, this suggests multifocal gliomas originating from a single tumor; by contrast, if the foci are widely separated, without demonstrable connecting pathways, they are likely multicentric gliomas, arising as independent tumors [2,14,15]. Various radiological features of multiple glioblastoma were described, characterizing the lesion as being located deep or outside the cortical–subcortical boundaries, being a solid nodule without central necrosis, and with an irregular shape, but not suggestive of metastasis. However, differentiating multicentric gliomas and multifocal gliomas based on radiological appearance is difficult.

In recent years, magnetic resonance imaging (MRI) permitted the identification of connections between neoplastic foci. For example, communication through association tracts was demonstrated as a continuous hyperintense area on T2-weighted imaging and fluid-attenuated inversion recovery (FLAIR) [3]. Unfortunately, as the underlying white matter invasion is invisible on conventional MRI, it is hard to identify anatomical connections between neoplastic foci [16]. Unlike conventional MR imaging, diffusion tensor imaging (DTI), combined with state-of-the-art probabilistic tractography algorithms, can help in detecting and quantifying this underlying tumor infiltration [17,18]. This non-invasive method allows the reconstruction of white matter pathways, connecting two regions at a voxel level [19], and, thus, may help to identify the otherwise invisible dissemination routes along fiber tracts between the neoplastic foci.

In this study, we used probabilistic fiber tracking with DTI to identify white matter pathways in patients with multiple gliomas, and then used cluster analysis to identify possible subgroups, based on the probabilistic values across white matter tracts between neoplastic foci. We then investigated the differences between the two subgroups in clinical data, selected molecular markers, and the results of other advanced MR imaging modalities, including diffusion-weighted imaging (DWI), proton MR spectroscopy (^1^H-MRS), and dynamic susceptibility contrast perfusion-weighted imaging (DSC-PWI).

## 2. Materials and Methods

### 2.1. Subjects

This retrospective data analysis study was approved by West China Hospital Ethics Committee (Chengdu, China), which waived the requirement to obtain individual informed consent. Data were selected from routine clinical scans carried out between February 2017 and February 2021. MRI scanning was conducted strictly, according to current clinical protocols, and no additional scans were carried out for purely research purposes. All patients whose data were used had newly diagnosed gliomas, and underwent resection or biopsy of the tumors. Based on modified Batzdorf criteria [2,20], only data from patients with at least two clearly separated foci at the time of initial MRI diagnosis were included. We excluded data from: (1) children and adolescents (≤18 years); (2) patients with evidence of cerebrospinal fluid spread or leptomeningeal dissemination; (3) patients who received any treatment such as radiation, chemotherapy, or surgery before MR examination; (4) studies with poor image quality, due to head motion or artifacts. Finally, data from 46 patients with multiple gliomas were included in the study cohort.

### 2.2. Molecular Analyses

For molecular biomarker analysis, tumor DNA was extracted from formalin-fixed and paraffin-embedded tissue samples, with a histologically estimated tumor cell content ≥80%. To assess mutation status, the genomic regions encompassing codons R132 of IDH1 and R172 of IDH2 were analyzed by pyrosequencing [21], using the forward primer 5′-TGATCCCCATAAGCAT-3′ and reverse primer 5′-CGACTGACACTATCGAT-3′ for IDH1, and forward primer 5′-AGCCCATCACCATTG-3′ and reverse primer 5′-TGCGATCGATCGCACGCA-3′ for IDH2. MGMT promoter methylation status analysis was performed with bisulfite sequencing, providing the percentage of methylated CG island in the sample. Nuclear ATRX status was determined by immunohistochemistry: >90% loss of nuclear staining for ATRX in tumor cells was considered positive for ATRX loss [22].

### 2.3. MRI Acquisition

Scans of all patients were acquired on a 3.0 T MRI equipped with a 20 channel phased-array head and neck coil (Skyra, Siemens Healthineers, Erlangen, Germany). The scanning protocol is presented in Table 1.

### 2.4. Image Processing

#### 2.4.1. DTI

The foci region, defined as the whole area of abnormal signal intensity on enhanced 3D-T1 and FLAIR, was manually segmented (by *, 12 years neuroradiology experience), using ITK-SNAP software (www.itksnap.org, accessed on 5 March 2021). The foci mask serves as the seed and target for probabilistic tractography. The DTI images were pre-processed using the FSL 6.0.2 Diffusion Toolbox (FDT) (http://fsl.fmrib.ox.ac.uk/fsl/fslwiki/FDT, accessed on 5 March 2021). Diffusion parameters at each whole-brain voxel were first estimated using BEDPOST [23]. For each subject, the resulting distributions were used for probabilistic fiber tracking using PROBTRACK [24], with FSL 6.0.2. This algorithm calculates white matter connectivity (streamlines) as an estimate of fiber connections between the seed and the target masks defined a priori (the neoplastic foci, segmented by ITK-SNAP) [25,26]. Finally, the probabilistic value, connectivity value, and fractional anisotropy (FA) between neoplastic foci were extracted from the probabilistic fiber tracking.

#### 2.4.2. Other Advanced MR Imaging Modalities

^1^H-MRS was analyzed using LCModel software [27]. The ratios of N-acetylaspartate (NAA)/creatine (Cr), choline (Cho)/Cr, lactate (Lac)/Cr, lipid (Lip)/Cr, and Cho/NAA in the multiple tumor foci were selected for further analysis. The CBV and relative CBV (rCBV, defined as the ratio of CBV of lesion to CBV of contralateral normal brain) were calculated, and the final values for each patient were the average over all foci. The foci mask with whole solid tumor was registered to the apparent diffusion coefficient (ADC) maps. The statistics tool of ITK-SNAP was used to obtain the mean ADC value of the whole foci mask. Then, the ADC of the lesion was compared with the ADC of the contralateral normal brain, to obtain relative ADC (rADC); the final values were the average of all foci. The details of these procedures are shown in Supplementary Figures S1 and S2.

### 2.5. Cluster Analysis

**Figure 1:**
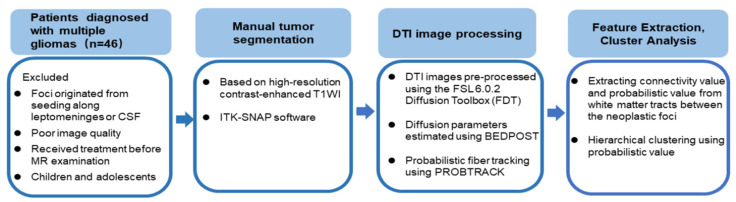
The workflow of probabilistic fiber tracking and cluster analysis.

Hierarchical clustering [28] was performed using the probabilistic value feature extracted from the probabilistic fiber tracking. The optimal cluster number was determined using Gap statistic [29], which compares the total intra-cluster variation for different values of k with their expected values under null reference distribution of the data (i.e., a distribution with no obvious clustering). Figure 1 shows this workflow.

T2WI and FLAIR images of each patient were reviewed by a radiologist (*, with 12 years neuroradiology experience). The presence of a continuous hyperintense area on T2WI or FLAIR images is generally accepted as indicating the presence of connection between the foci [30].

### 2.6. Inter-Subgroup Comparison

After identification of subgroups by cluster analysis, the inter-subgroup differences were explored. Differences in age were compared using Student’s *t*-test. Sex distribution, molecular status including isocitrate dehydrogenase mutation (IDHmut), O^6^-methylguanine-methyltransferase promoter methylation (MGMTmet), alpha thalassemia/mental retardation syndrome X-linked loss (ATRXloss), and WHO glioma grades were compared using the chi-square test. The Ki-67-labeled proliferation index was compared using the 2-tailed *t* test. The FA and measures derived from ^1^H-MRS, PWI, and DWI were compared using the Mann–Whitney U test. All statistical test results were considered significant if *p* < 0.05 (FDR-corrected).

As distance may affect the connectivity value, we used the Euclidean distance to measure the spatial distance between two foci, implemented as a heuristic of white matter paths:

$$d=\sqrt{{\left(x_{2}-x_{1}\right)}^{2}+{\left(y_{2}-y_{1}\right)}^{2}+{\left(z_{2}-z_{1}\right)}^{2}}$$

in which *x*, *y*, and *z* represent the three-dimensional Montreal Neurological Institute (MNI) coordinate located in the core of the two foci (see Supplementary Figure S3). Then, we conducted a general linear model, to compare the connectivity value between the subgroups, using Euclidean distance as the covariate.

## 3. Results

### 3.1. Demographic and Clinical Characteristics of the Patients

The study includes 46 patients (25 males and 21 females) with multiple gliomas. Median age at the time of diagnosis is 42 years (range 20–76 years). Intracerebral masses comprise 17 cases of glioblastomas (WHO grade IV), 2 cases of H3K27M mutant diffuse midline glioma (WHO grade IV), 7 cases of anaplastic astrocytoma (WHO grade III), 6 cases of oligodendroglioma (WHO grade II), and 14 cases of diffuse astrocytoma (WHO grade II).

### 3.2. Probabilistic Fiber Tracking

The mean probabilistic value extracted from fibers between neoplastic foci of all patients is 0.23 ± 0.22, the mean connectivity value is 18,967 ± 2251, and the mean FA of the fibers is 0.32 ± 0.11.

### 3.3. Hierarchical Clustering

**Figure 2:**
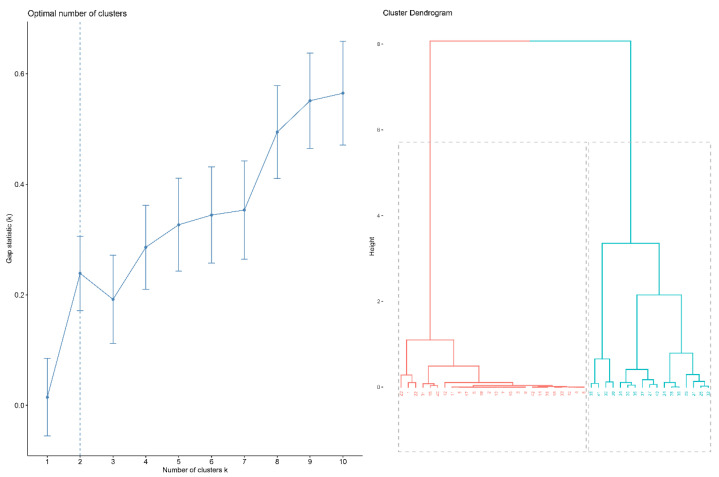
Cluster analysis. (**left**) The optimal number of clusters; (**right**) dendrogram illustration of hierarchical clustering.

The result of hierarchical clustering analysis is shown as a dendrogram in Figure 2. The estimate of the optimal clusters is the value that maximizes the gap statistic, at which point the clustering structure is maximally distant from the random uniform distribution. The gap statistic plot (Figure 2) shows the statistics by number of clusters (k), with standard errors shown as vertical segments, and the optimal value of k marked with the vertical dashed blue line: k = 2 in this dataset (Figure 2).

**Figure 3:**
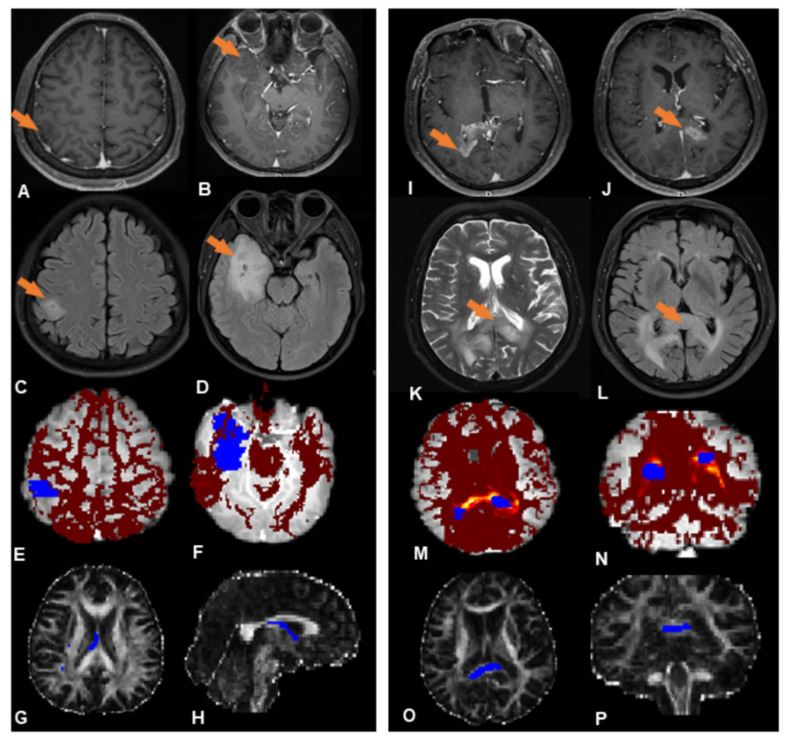
Illustrative PROBTRACK results from each subgroup. ((**A**–**H**) (left panel) Subgroup 1, (**I**–**P**) (right panel) Subgroup 2). (**A**,**B**) Two neoplastic foci (orange arrows) at the right temporal lobe and parietal lobe exhibit slight enhancement in T1-weighted MR images. (**C**,**D**) No abnormal FLAIR signal between the foci. (**E**,**F**) White matter connectivity between lesions derived from probabilistic fiber tracking (blue regions are the two neoplastic foci; the red region is the likelihood of a path between them). (**G**,**H**) The probabilistic path extracted from the probabilistic fiber tracking (blue region). (**I**,**J**) Two neoplastic foci (orange arrows) at the splenium of corpus callosum exhibit obvious enhancement in T1-weighted images; (**K**,**L**) A continuous hyperintense area on T2WI and FLAIR between the foci. (**M**,**N**) White matter connectivity between neoplastic foci derived from probabilistic fiber tracking (blue regions are the neoplastic foci, the ‘hot’ region is the likelihood of a path existing between them). (**O**,**P**) Probabilistic path extracted from the probabilistic fiber tracking (blue region).

Subsequent analysis mainly focuses on these two subgroups (subgroups 1 and 2, indicated in red and blue, respectively, in Figure 2); 19 patients (41%) were placed in subgroup 1, and 27 patients (59%) in subgroup 2. Figure 3 shows, illustratively, the PROBTRACK results of two selected patients, one from each subgroup.

The radiologist reviewed the T2WI and FLAIR images of each patient in the subgroups: in subgroup 1, there is no abnormal signal observed between the foci, while in subgroup 2 all except three patients show contiguous abnormal signal between the foci (Supplementary Figure S4).

Combining the cluster analysis with visual assessment, subgroup 1 shows a trend towards widely separated lesions with no anatomical connections, which may be termed the true multicentric gliomas, while subgroup 2 shows anatomical continuity between lesions, which may be termed the multifocal gliomas [31].

### 3.4. Subgroup Differences

Table 2 shows the demographic, histopathological, and MRI/MRS characteristics of the two subgroups. The subgroups do not differ with respect to age or sex distribution, nor in the status of IDHmut and ATRX loss, the Ki-67-labeled proliferation index, or WHO grades. Subgroup 1 comprises 10 glioblastomas, 2 anaplastic astrocytomas, 1 oligodendroglioma, and 6 diffuse astrocytomas; subgroup 2 comprises 7 glioblastomas, 2 H3K27M mutant diffuse midline gliomas, 5 anaplastic astrocytomas, 5 oligodendrogliomas, and 8 diffuse astrocytomas.

The average white matter connectivity between multiple foci of subgroup 1 is significantly less than in subgroup 2. The subgroups show no significant differences in FA.

**Figure 4:**
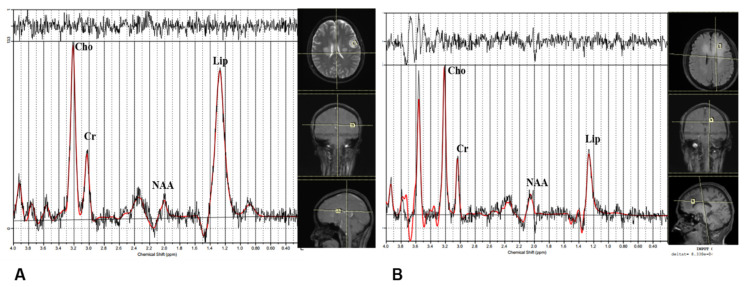
Illustrative results of analysis of magnetic resonance spectroscopy data. (**A**) A patient from subgroup 1. (**B**) A patient from subgroup 2.

By ^1^H-MRS, there is no significant difference between the subgroups in NAA/Cr, Cho/Cr, Lac/Cr, or Cho/NAA. The ratio Lip/Cr is significantly higher in subgroup 1: Figure 4 shows illustrative spectral data analysis from the two subgroups.

**Figure 5:**
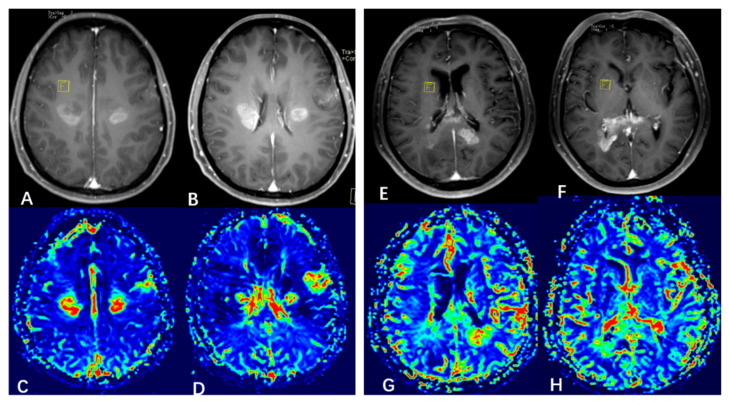
Illustrative cerebral blood volume (CBV) maps from the two subgroups. ((**A**–**D**) (left panel) subgroup 1, (**E**–**H**) (right panel) subgroup 2). (**A**,**B**) Enhanced T1-weighted MRI showing 3 neoplastic foci. (**C**,**D**) CBV map showing elevated CBV at the foci. (**E**,**F**) Enhanced T1-weighted MRI showing 2 neoplastic foci. (**G**,**H**) CBV map showing elevated CBV at the foci.

**Figure 6:**
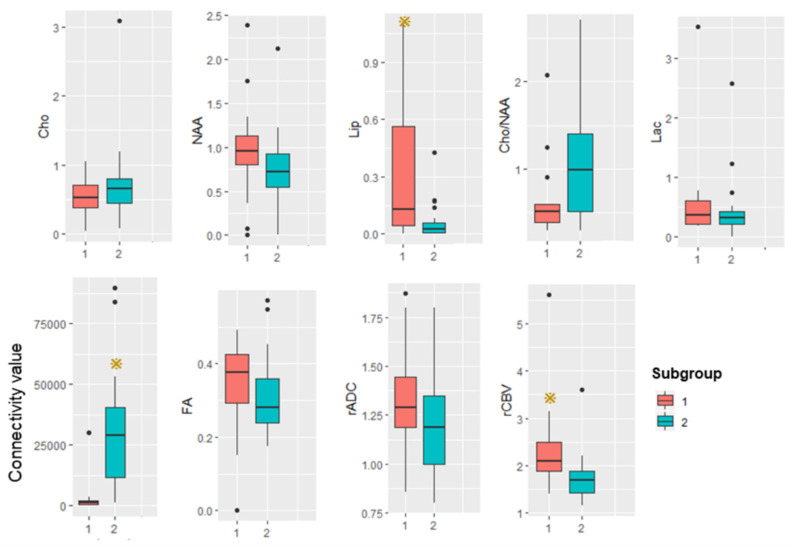
Box plot summarizing advanced MRI measurements in the two subgroups. Yellow star represents *p* is less than 0.05.

By DSI-PWI and DWI, mean rCBV is significantly higher in subgroup 1 than subgroup 2. Figure 5 shows illustrative CBV maps from the two subgroups. There are no significant differences in mean rADC between the subgroups. Figure 6 summarizes the advanced MR imaging measurements as box plots.

## 4. Discussion

Many studies address the classification of multiple gliomas, seeking to define different subgroups based on origin and growth pattern. For example, multifocal and multicentric gliomas are defined based on physical connection between the foci [20,32,33]: multifocal gliomas result from dissemination or spreading via established pathways, such as white matter fibers and cerebrospinal fluid, or local metastasis; in contrast, multicentric gliomas exhibit widely separated foci in different lobes or hemispheres, whose distribution cannot be explained by any of these pathways or mechanisms [2,34]. As conventional MRI is unable to delineate microscopic tumor infiltration along white matter fibers [35], it is not useful for making this distinction. Here, we used probabilistic fiber tractography with DTI to detect subtle white matter connections between the foci, hoping, thereby, not only to aid the identification of multicentric vs. multifocal gliomas, but also provide new insight into the nature of this tumor.

Using cluster analysis, we identify two distinct patterns of white matter connection in multiple gliomas: increased and decreased connectivity of the inter-focal white matter. In methodological terms, this connectivity quantifies the likelihood of a path between the seed focus and target focus: the higher the connectivity, the greater the likelihood of connection [26]. In subgroup 1, the smaller inter-focal connectivity indicates weak white matter linking, tending to suggest independent origin; in subgroup 2, the relative greater connectivity suggests that the multiple foci develop through metastasis along white matter pathways, and it is tempting to speculate that the neoplastic foci are, therefore, of common origin and may share the same genetic aberrations. It is worth noting that in subgroup 2, all except three patients show contiguous abnormal signal between the foci. After carefully checking the location, distance, and foci volume of these three exceptions, the main difference is that their probability values are much larger than the mean value of subgroup 1. This may suggest that glioma can spread along white matter tracts that cannot be identified visually. If so, then cluster analysis, based on probabilistic fiber tracking, may be a more reliable tool to evaluate whether there are microscopic connections between foci. These findings should, of course, be viewed as preliminary, but do suggest a direction for future research.

Notably, the two subgroups do not differ in molecular characteristics (IDHmut, ATRX loss, MGMTmet, and Ki-67-labeled proliferation index) or WHO grades. In contrast, a previous study of 14 cases of multicentric glioma finds no IDH1 mutation or ATRX loss [36]. An earlier study of 10 cases (predating modern molecular methods) finds multicentric gliomas to be histologically of low-grade, with gradual evolution, whereas multifocal gliomas are of high-grade malignancy [37]. These differences are likely due to the limitations that prior studies acknowledged: possible false negative results due to lack of DNA sequencing for IDH1 mutation or ATRX loss, and relatively small sample size [36].

^1^H-MRS, DSC-PWI, and DWI provide information on metabolism, vascularity, and cellularity, respectively. Subgroup 1 has a higher rCBV and Lip/Cr ratio than subgroup 2. As both these findings can result from rapid tumor growth, related to a higher tumor grade [38,39], this might suggest that foci of independent origin in multicentric glioma tend to have higher vascularity and more intratumoral necrosis. However, there are no significant differences in WHO grades between the two subgroups. Future studies, with a larger number of patients, should investigate this further.

Thus, our study reveals two distinct subgroups of multiple gliomas, based on the probabilistic fiber tracking and cluster analysis. This finding has some practical significance: while the foci of multifocal glioma, which are homologous tumors, can be treated with the same therapeutic strategy, the foci of multicentric glioma tend to have different origins and, potentially, biologic characteristics, so focus-based treatment planning is required. A previous study shows that the median survival in patients with multicentric glioblastomas treated with aggressive multiple-craniotomy resection of all lesions is 12.9 months, compared to 9.6 months in patients with multifocal disease [40]. Thus, differentiating multicentric gliomas from multifocal gliomas has practical clinical value, and evidence from probabilistic fiber tractography may provide information for the choice of surgical approach, and the estimation of prognosis.

This study has several limitations. Firstly, although the patient population is larger than previous studies, it may still be insufficient to fully reveal the heterogeneity of multiple gliomas. Further studies, with larger sample sizes and using a higher order model, are therefore needed to validate our findings, and improve the identification of subgroups of multiple gliomas. Secondly, this is a single-center study and requires validation with data from multiple centers. Finally, most patients included have two foci, while only two patients have three foci; sub-analysis taking into account number of foci will be valuable in future studies.

## 5. Conclusions

Using cluster analysis, we identified two subgroups of patients with multiple gliomas having low and high probabilistic connections of white matter. Multiple gliomas, with smaller inter-focal connectivity, tend to have independent origins, i.e., are true multicentric glioma; other gliomas, with greater connectivity, tend to share a common origin and develop through early white matter spreading. The two categories of multiple gliomas differ in some metabolic and vascularity characteristics. These findings may be helpful for preoperative evaluation and personalized treatment planning.

## Figures and Tables

**Table 1:** MRI acquisition protocol.

Parameters	MPRAGE (3D T1WI)	Enhanced 3D T1WI	T2WI	FLAIR	DWI	DSC-PWI	^1^H-MRS
Repetition time (ms)	1630	1630	4500	6000	6000	1640	2000
Echo time (ms)	2.3	2.3	105	81	93	30	135
Flip angle	8°	8°	150°	90°	90°	90°	150°
Slice thickness (mm)	1	1	5	5	3	5	5
In-plane resolution (mm)	0.5 × 1	0.5 × 1	0.5 × 0.5	0.7 × 0.7	1.8 × 1.8	1.7 × 1.3	0.5 × 0.5
Acquisition time (s)	187	200	58	62	206	105	394
Directions	-	-	-	-	30	-	-

Abbreviations: MPRAGE, magnetization-prepared rapid acquisition gradient echo.

**Table 2:** Demographic, histopathological, and MR imaging and spectroscopy results in the two subgroups.

Characteristic	Subgroup		Statistical Analysis	
	1	2	Statistic	*p*
*Demographics*				
Age (y)	42.3 ± 14.2	41.8 ± 13.2	0.027	0.98
Sex (male/female)	8/11	17/10	1.96	0.16
*Histopathology*				
Low grade (II)	7 (37%)	13 (48%)	0.58	0.45
High grade (III + IV)	12 (63%)	14 (52%)	-	-
IDHmut (with/without)	10/9	11/16	0.64	0.44
MGMTmet (with/without)	12/7	10/17	3.05	0.081
ATRX loss (with/without)	9/10	12/15	0.038	0.85
Ki-67 proliferation index	0.15 ± 0.12	0.20 ± 0.16	−1.07	0.29
*MR imaging*				
Connectivity	2675 ± 1098	30432 ± 22707	−5.23	*p* = 0.000016 *
FA	0.34 ± 0.116	0.30 ± 0.108	1.15	0.26
rCBV	2.31 ± 0.95	1.73 ± 0.48	−3.11	0.002 *
rADC	1.32 ± 0.25	1.21 ± 0.27	−1.58	0.12
*MR spectroscopy*				
Cho/Cr	0.52 ± 0.26	0.74 ± 0.57	−1.49	0.14
NAA/Cr	0.96 ± 0.62	0.77 ± 0.41	−1.43	0.14
Cho/NAA	1.07 ± 0.54	1.34 ± 0.42	−1.49	0.13
Lip/Cr	0.32 ± 0.22	0.060 ± 0.051	−2.71	0.006 *
Lac/Cr	0.62 ± 0.85	0.45 ± 0.32	−0.72	0.47

Results are mean ± SD or numbers in two categories (x/y). Abbreviations: IDHmut, isocitrate dehydrogenase mutation; MGMTmet, O6-methylguanine-methyltransferase promoter methylation; ATRX loss: alpha thalassemia/mental retardation syndrome X-linked loss; FA, fractional anisotropy; rCBV, relative cerebral blood volume; NAA, N-acetylaspartate; Cr, creatine; Cho, choline; Lac, lactate; Lip, lipid; and rADC, relative apparent diffusion coefficient. * *p* is less than 0.05.

## Data Availability

The data presented in this study are available on request from the corresponding author. The data are not publicly available due to the privacy of participants.

## Supplementary Materials

The following supporting information can be downloaded at: https://www.mdpi.com/article/10.3390/jcm11133693/s1, Figure S1: ROI placement from a representative case. Figure S2: The procedure to calculate the ADC value. Figure S3: Measuring Euclidean distance in a representative case. Figure S4. Patients show contiguous abnormal signal between the foci in the subgroup 2.

## References

[1] Barnard R.O., Geddes J.F. (1987). The incidence of multifocal cerebral gliomas. A histologic study of large hemisphere sections. Cancer.

[2] Batzdorf U., Malamud N. (1963). the problem of multicentric gliomas. J. Neurosurg..

[3] Djalilian H.R., Shah M.V., Hall W.A. (1999). Radiographic incidence of multicentric malignant gliomas. Surg. Neurol..

[4] Showalter T.N., Andrel J., Andrews D.W., Curran W.J., Daskalakis C., Werner-Wasik M. (2007). Multifocal glioblastoma multiforme: Prognostic factors and patterns of progression. Int. J. Radiat. Oncol. Biol. Phys..

[5] Thomas R.P., Xu L.W., Lober R.M., Li G., Nagpal S. (2013). The incidence and significance of multiple lesions in glioblastoma. J. Neurooncol..

[6] Patil C.G., Yi A., Elramsisy A., Hu J., Mukherjee D., Irvin D.K., Yu J.S., Bannykh S.I., Black K.L., Nuno M. (2012). Prognosis of patients with multifocal glioblastoma: A case-control study. J. Neurosurg..

[7] Liu Q., Liu Y., Li W., Wang X., Sawaya R., Lang F.F., Yung WK A., Chen K., Fuller G.N., Zhang W. (2015). Genetic, epigenetic, and molecular landscapes of multifocal and multicentric glioblastoma. Acta Neuropathol..

[8] Abou-El-Ardat K., Seifert M., Becker K., Eisenreich S., Lehmann M., Hackmann K., Rump A., Meijer G., Carvalho B., Temme A. (2017). Comprehensive molecular characterization of multifocal glioblastoma proves its monoclonal origin and reveals novel insights into clonal evolution and heterogeneity of glioblastomas. Neuro-Oncol..

[9] Krex D., Mohr B., Appelt H., Schackert H.K., Schackert G. (2003). Genetic analysis of a multifocal glioblastoma multiforme: A suitable tool to gain new aspects in glioma development. Neurosurgery.

[10] Akimoto J., Sasaki H., Haraoka R., Nakajima N., Fukami S., Kohno M. (2014). Case of radiologically multicentric but genetically identical multiple glioblastomas. Brain Tumor Pathol..

[11] Kim J., Lee I.-H., Cho H.J., Park C.-K., Jung Y.-S., Kim Y., Nam S.H., Kim B.S., Johnson M.D., Kong D.-S. (2015). Spatiotemporal Evolution of the Primary Glioblastoma Genome. Cancer Cell.

[12] Fares Y., Younes M., Kanj A., Barnes P.R., Muñiz J. (2009). Multicentric glioma: Problems & interpretations [corrected]. P R Health Sci. J..

[13] Lombardi G., Della Puppa A., Gardiman M.P., Rossi S., Candiotto C., Zanatta L., Bertorelle R., De Rossi A., Fassan M., Zagonel V. (2018). Discordance of IDH mutational status between lesions in an adult patient with multifocal glioma. Neuro-Oncol..

[14] Arcos A., Romero L., Serramito R., Santin J.M., Prieto A., Gelabert M., Arraez M.A. (2012). Multicentric glioblastoma multiforme. Report of 3 cases, clinical and pathological study and literature review. Neurocirugia.

[15] Van Tassel P., Lee Y.Y., Bruner J.M. (1988). Synchronous and metachronous malignant gliomas: CT findings. AJNR Am. J. Neuroradiol..

[16] Painter K.J., Hillen T. (2013). Mathematical modelling of glioma growth: The use of Diffusion Tensor Imaging (DTI) data to predict the anisotropic pathways of cancer invasion. J. Theor. Biol..

[17] Mohan S., Wang S., Coban G., Kural F., Chawla S., O’Rourke D.M., Poptani H. (2019). Detection of occult neoplastic infiltration in the corpus callosum and prediction of overall survival in patients with glioblastoma using diffusion tensor imaging. Eur. J. Radiol..

[18] Price S.J., Peña A., Burnet N.G., Pickard J.D., Gillard J.H. (2004). Detecting glioma invasion of the corpus callosum using diffusion tensor imaging. Br. J. Neurosurg..

[19] Johansen-Berg H., Behrens T.E. (2006). Just pretty pictures? What diffusion tractography can add in clinical neuroscience. Curr. Opin. Neurol..

[20] Shakur S.F., Bit-Ivan E., Watkin W.G., Merrell R.T., Farhat H.I. (2013). Multifocal and multicentric glioblastoma with leptomeningeal gliomatosis: A case report and review of the literature. Case Rep. Med..

[21] Setty P., Hammes J., Rothämel T., Vladimirova V., Kramm C.M., Pietsch T., Waha A. (2010). A pyrosequencing-based assay for the rapid detection of IDH1 mutations in clinical samples. J. Mol. Diagn..

[22] Ikemura M., Shibahara J., Mukasa A., Takayanagi S., Aihara K., Saito N., Aburatani H., Fukayama M. (2016). Utility of ATRX immunohistochemistry in diagnosis of adult diffuse gliomas. Histopathology.

[23] Behrens T.E.J., Woolrich M.W., Jenkinson M., Johansen-Berg H., Nunes R.G., Clare S., Matthews P.M., Brady J.M., Smith S.M. (2003). Characterization and propagation of uncertainty in diffusion-weighted MR imaging. Magn. Reson. Med..

[24] Behrens T.E., Berg H.J., Jbabdi S., Rushworth M.F., Woolrich M.W. (2007). Probabilistic diffusion tractography with multiple fibre orientations: What can we gain?. Neuroimage.

[25] Shott M.E., Pryor T.L., Yang T.T., Frank G.K.W. (2016). Greater Insula White Matter Fiber Connectivity in Women Recovered from Anorexia Nervosa. Neuropsychopharmacol. Off. Publ. Am. Coll. Neuropsychopharmacol..

[26] Segobin S., Laniepce A., Ritz L., Lannuzel C., Boudehent C., Cabé N., Urso L., Vabret F., Eustache F., Beaunieux H. (2019). Dissociating thalamic alterations in alcohol use disorder defines specificity of Korsakoff’s syndrome. Brain.

[27] Provencher S.W. (1993). Estimation of metabolite concentrations from localized in vivo proton NMR spectra. Magn. Reson. Med..

[28] Johnson S.C. (1967). Hierarchical clustering schemes. Psychometrika.

[29] Tibshirani R., Walther G., Hastie T. (2002). Estimating the Number of Clusters in a Data Set via the Gap Statistic. J. R. Statist. Soc. B.

[30] Di Carlo D.T., Cagnazzo F., Benedetto N., Morganti R., Perrini P. (2019). Multiple high-grade gliomas: Epidemiology, management, and outcome. A systematic review and meta-analysis. Neurosurg. Rev..

[31] di Russo P., Perrini P., Pasqualetti F., Meola A., Vannozzi R. (2013). Management and outcome of high-grade multicentric gliomas: A contemporary single-institution series and review of the literature. Acta Neurochir..

[32] Salvati M., Oppido P.A., Artizzu S., Fiorenza F., Puzzilli F., Orlando E.R. (1991). Multicentric gliomas. Report of seven cases. Tumori.

[33] Budka H., Podreka I., Reisner T., Zeiler K. (1980). Diagnostic and pathomorphological aspects of glioma multiplicity. Neurosurg. Rev..

[34] Giannopoulos S., Kyritsis A.P. (2010). Diagnosis and management of multifocal gliomas. Oncology.

[35] Delgado A.F., Fahlstrom M., Nilsson M., Berntsson S.G., Zetterling M., Libard S., Alafuzoff I., van Westen D., Latt J., Smits A. (2017). Diffusion Kurtosis Imaging of Gliomas Grades II and III—A Study of Perilesional Tumor Infiltration, Tumor Grades and Subtypes at Clinical Presentation. Radiol. Oncol..

[36] Karlowee V., Amatya V.J., Hirano H., Takayasu T., Nosaka R., Kolakshyapati M., Yoshihiro M., Takeshima Y., Sugiyama K., Arita K. (2017). Multicentric Glioma Develops via a Mutant IDH1-Independent Pathway: Immunohistochemical Study of Multicentric Glioma. Pathobiology.

[37] Jomin M., Lesoin F., Lozes G., Delandsheer J.M., Biondi A., Krivosic I. (1983). Multifocal glioma. Apropos of 10 cases. Neuro-Chirurgie.

[38] Oshiro S., Tsugu H., Komatsu F., Abe H., Onishi H., Ohmura T., Iwaasa M., Sakamoto S., Fukushima T. (2007). Quantitative assessment of gliomas by proton magnetic resonance spectroscopy. Anticancer Res..

[39] Law M., Yang S., Babb J.S., Knopp E.A., Golfinos J.G., Zagzag D., Johnson G. (2004). Comparison of cerebral blood volume and vascular permeability from dynamic susceptibility contrast-enhanced perfusion MR imaging with glioma grade. AJNR Am. J. Neuroradiol..

[40] Hassaneen W., Levine N.B., Suki D., Salaskar A.L., de Moura Lima A., McCutcheon I.E., Prabhu S.S., Lang F.F., DeMonte F., Rao G. (2011). Multiple craniotomies in the management of multifocal and multicentric glioblastoma. J. Neurosurg..

