# Supplementary material for: Two Patterns of White Matter Connection in Multiple Gliomas: Evidence from Probabilistic Fiber Tracking
Source: J Clin Med. 2022 Jun 27;11(13):3693. doi: 10.3390/jcm11133693 (PMC9267772; doi:10.3390/jcm11133693)
Supplement: Supplementary file 1 [file jcm-11-03693-s001.zip › jcm-1688849-supplementary.pdf]

Supplementary Material

Figure S1. ROI placement from a representative case.

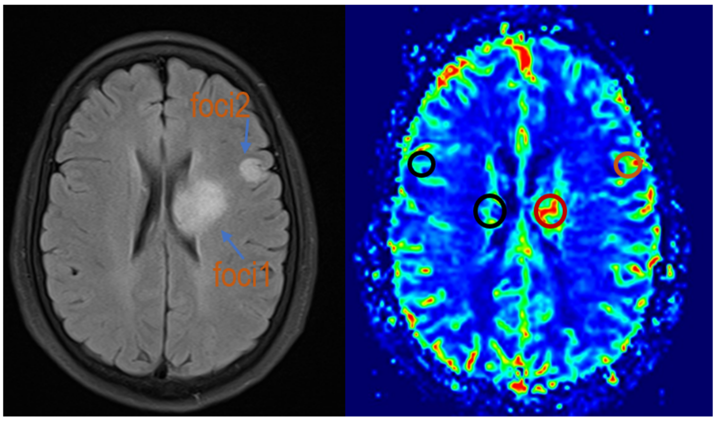

The ROI in the tumor was manually placed in the hot color (high perfusion) regions within the solid portions with the color CBV map overlaid. Then the ROI in the normal tissue was placed in the contralateral normal-appearing brain. Cystic, necrotic or hemorrhagic parts of the lesion and adjacent bone, air and blood vessels were avoided.

Figure S2. The procedure to calculate the ADC value.

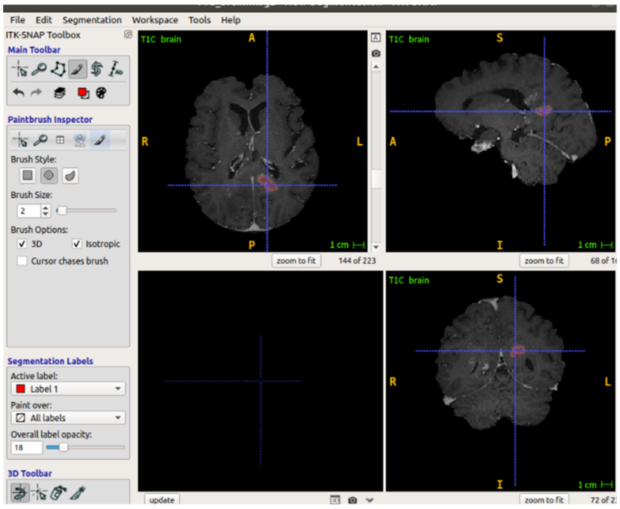	Step 1: Open "ITK-SNAP", click "Load Main Image" button to load the enhanced-3DT1 images, then click "brush"" button to manually slice-by-slice segment the foci 1 (red region).<br><br>Similarly, use "brush"" button to manually slice-by-slice segment the foci 2 (green region).

<table>
<tr><td>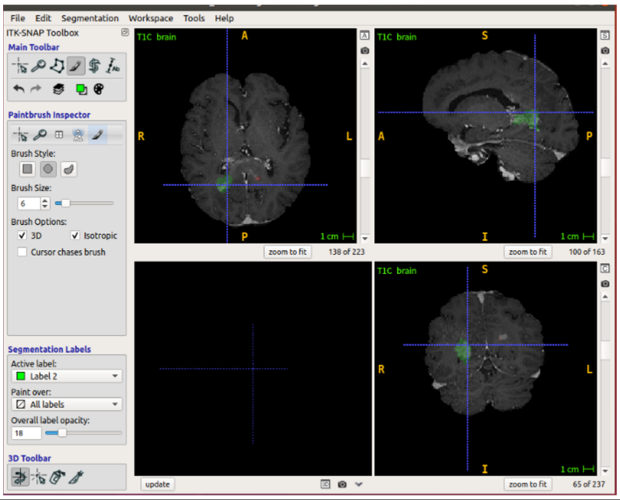
</td><td></td></tr>
<tr><td>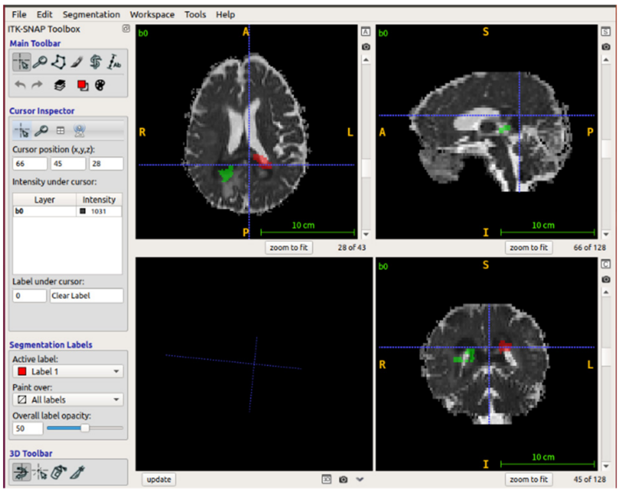
</td><td>Step 2: Use fsl flirt to register the masks of foci 1(red region) foci 2(green region) to the ADC map.</td></tr>
<tr><td>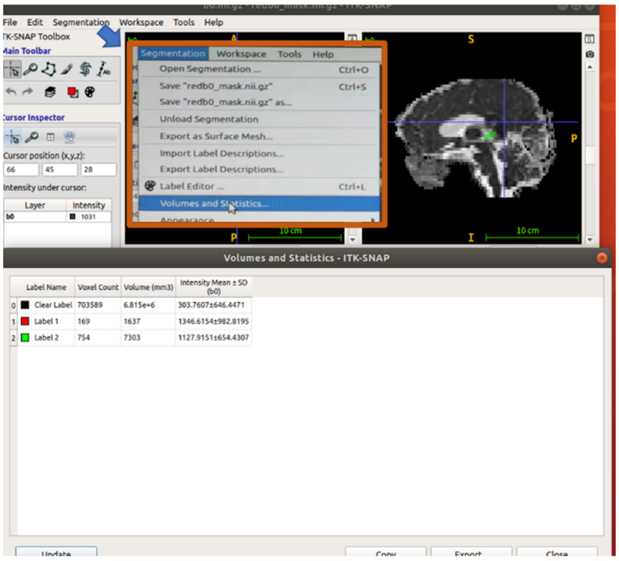
</td><td>Step 3. Click “volumes and Statistics” button to obtain the mean ADC value of the whole segmentation mask.</td></tr>
<tr><td></td><td></td></tr>
</table>

Figure S3 Measuring Euclidean distance in a representative case.

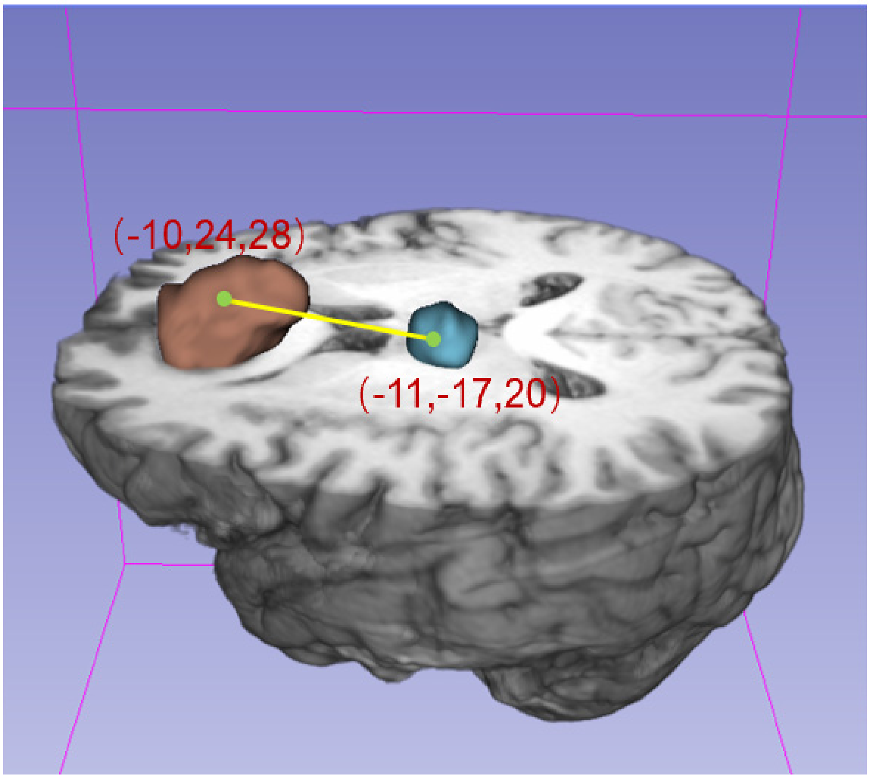

Figure S4.

Three patients show contiguous abnormal signal between the foci in the subgroup 2. Subfigures **A, B, C** represents above patients respectively.

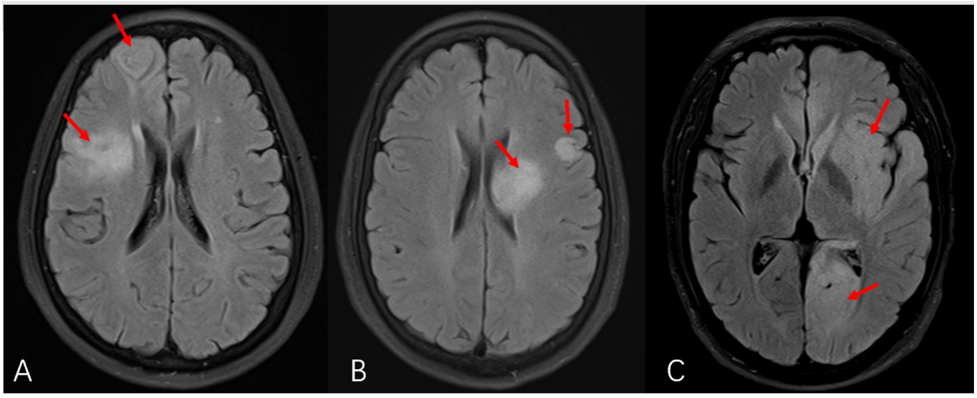

Patient ID	Location		Volume（mm3）		Euclidean distance	Probabilistic value
	Foci1	Foci2	Foci1	Foci2		
Patient6 (figure A)	Right frontal lobe	Right frontal lobe	7524	3240	52.13	.2674768
Patient14 (figure B)	Left basal ganglia	Left frontal lobe	3664	1118	34.48	.2113550
Patient22 (figure C)	Left Temporal lobe	Left Occipital lobe	1.122e+4	6055	78.89	.1974768
